# Supplementary material for: Ancient proteins from ceramic vessels at Çatalhöyük West reveal the hidden cuisine of early farmers
Source: Nat Commun. 2018 Oct 3;9:4064. doi: 10.1038/s41467-018-06335-6 (PMC6170438; doi:10.1038/s41467-018-06335-6)
Supplement: Supplementary file 1 — Supplementary Information [file 41467_2018_6335_MOESM1_ESM.pdf]

Supplementary Information

Ancient proteins in ceramic vessels from Çatalhöyük West reveal the hidden cuisine of early farmers.

Jessica Hendy, Andre C. Colonese, Ingmar Franz, Ricardo Fernandes, Roman Fischer, David Orton, Alexandre Lucquin, Luke Spindler, Jana Anvari, Elizabeth Stroud, Peter F. Biehl, Camilla Speller, Nicole Boivin, Meaghan Mackie, Rosa R. Jersie-Christensen, Jesper V. Olsen, Matthew J. Collins, Oliver E. Craig, Eva Rosenstock

This PDF contains:

- **Supplementary Table 1.** Sample codes, morphologically determined vessel types and weights of sample used for protein and lipid extractions.
- **Supplementary Table 2.** Summary of lipid extraction and isotopic analysis
- **Supplementary Table 3.** Regression equations for $\delta^{13}C_{18:0}$ against $\delta^{13}C_{16:0}$ in extracts of modern reference fats and oils
- **Supplementary Table 4.** Summary of lipid extraction and isotopic analysis from calcified deposit, inner wall of ceramic matrix, and outer wall of ceramic matrix.
- **Supplementary Figure 1.** Sample locations of pottery sherds analyzed in this study.
- **Supplementary Figure 2.** Relationship between total weight contributions of plant vs. dairy foods and their FA contributions.
- **Supplementary Figure 3.** Comparison of summarized results from lipid and protein analysis on ceramic matrix and calcified deposit samples
- **Supplementary Figure 4.** Partial total ion chromatogram of typical lipid extracts from calcite (CW18, CW22) and ceramic matrices (CW18 and CW22).
- **Supplementary References**

Supplementary Table 1. Sample codes, morphologically determined vessel types and weights of sample used for protein and lipid extractions. The asterisk denotes samples which have painted exteriors. NA denotes that the experiment was not performed owing to insufficient material for multiple analyses.

Sample ID	Vessel Type	Protein Analysis #1 (mg)	Protein Analysis #2 (mg)	Lipid Extraction (mg)
		Calcified Deposit		
CW8	"S-Profile" Bowl	66.4	51.0	126.97
CW10	Bowl	67.8	51.0	170
CW11	Bowl or Jar	51.7	77.2	203.2
CW18	Possible Bowl	59.5	69.5	363.18
CW20	"S-Profile" Bowl	54.8	NA	69.31
CW21	"S-Profile" Bowl	72.6	59.5	151.93
CW22	Possible Jar	63.2	NA	120.7
CW23	"S-Profile" Bowl	49.1	51.6	NA
CW24	"S-Profile" Bowl	65.4	53.6	88.16
CW27	Bowl	51.9	54.3	279.04
CW28 (Burnt)	Bowl	16.9	NA	NA
		Ceramic Matrix (Inner Wall)		
CW10	Bowl	51.4	48.7	1196.5
CW18	Possible Bowl	NA	46.3	664.53
CW20	"S-Profile" Bowl	49.0	60.1	665.54
CW22	Possible Jar	51.3	56.8	526.22
CW24	"S-Profile" Bowl	44.5	32.7	526.22
		Ceramic Matrix (Outer Wall)		
CW10*	Bowl	46.1	56.0	621.55
CW20*	"S-Profile" Bowl	49.6	50.6	284.3
CW22	Possible Jar	42.8	56.5	205.26

Supplementary Table 2. Summary of lipid extraction and isotopic analysis.

Sample	Total lipid (µg/g) (Acid Extraction)	$\delta^{13}C_{16:0}$	$\delta^{13}C_{18:0}$	$\Delta^{13}C$ ($C_{16:0}$-$C_{18:0}$)	Interpretation
			Calcified Deposit		
CW8	109.24	-24.66	-27.23	-2.57	Ruminant
CW10	9,36	-24.12	-27.11	-2.99	Ruminant
CW11	357.48	-26.57	-28.29	-1.72	Ruminant
CW18	489.48	-26.23	-28.68	-2.46	Ruminant
CW20	34.61	-28.18	-28.62	-0.44	Ruminant
CW21	34.59	-26.7	-28.45	-1.74	Ruminant
CW22	436.67	-26.86	-30.5	-3.64	Ruminant Dairy
CW24	87.09	-26.3	-28.3	-2	Ruminant
CW27	23.25	-25.9	-28.05	-2.15	Ruminant
			Ceramic Matrix (Inner Wall)		
CW10	46.09	-29.05	-29.28	-0.23	Non-Ruminant
CW11	732.84	-29.28	-29.34	-0.05	Non-Ruminant
CW18	522.13	-28.46	-29.41	-0.95	Non-Ruminant
CW20	135.27	-29.5	-29.45	0.06	Non-Ruminant
CW22	2195.88	-27.24	-31.36	-4.13	Ruminant Dairy
CW24	41.23	-26.9	-28.84	-1.93	Ruminant
			Ceramic Matrix (Outer Wall)		
CW10	16.61	-29.15	-29.09	0.06	Non-Ruminant
CW20	19428.71	-29.94	-29.52	0.42	Non-Ruminant
CW22	142.96	-29.36	-29.66	-0.3	Non-Ruminant

Supplementary Table 3. Regression equations for $\delta^{13}C_{18:0}$ against $\delta^{13}C_{16:0}$ in extracts of modern reference fats and oils

Source	n	Gradient (± σ)	Intercept (± σ)	R^2
Plant	61	0.87 (0.04)	-3.72 (1.63)	0.83
Ruminant Dairy	72	0.95 (0.04)	-6.10 1.17)	0.87
Ruminant Adipose	85	0.95 (0.02)	-3.01 (0.54)	0.86

Supplementary Table 4. Summary of lipid extraction and isotopic analysis from calcified deposit (*), inner wall of ceramic matrix (**), and outer wall of ceramic matrix (***). Abbreviations: SFA, saturated fatty acid; TMTD, 4,8,12-trimethyltridecanoic acid; MUFA, monounsaturated fatty acid; PUFA, polyunsaturated fatty acid; BCFA, branched fatty acid; ALD, Aldehyde; K, mid-chain ketones; ALK, *n*-alkanes; ALC, *n*-alcohols; D, diacids; 10-oxo; 10-oxo-octadecanoic acid; HFA, Hydroxy fatty acids; CH, cholesterol, Phy, phytanic acid and %SRR[1]; Pri, pristanic acid.

Sample ID	AE - Lipid concentration (μg/g)	TLE - Lipid concentration (μg/g)	Lipid detected (AE)	Lipid detected (TLE)	$\delta^{13}C_{C16:0}$	$\delta^{13}C_{C18:0}$	$\Delta^{13}C$ ($C_{16:0}$-$C_{18:0}$)	Isotope-derived attribution
*CW8	109.24	-	SFA (C8:0 – C30:0), MUFA (C16:1, C18:1, C20:1, C21:1, C22:1, C24:1), PUFA (C18:2), BCFA (C14:0, C15:0, C17:0, C23:0), ALK (C11, C27), CH, 10-oxo, ALD (C14, C16, C18), ALK, D (C8:0, C9:0), Phy (60.4%), Pri, TMDT.	-	-24.66	-27.23	-2.57	Ruminant
*CW10	9.36	-	SFA (C12:0 – C32:0), MUFA (C18:1, C20:1, C22:1, C24:1), PUFA (C18:2), BCFA (C14:0 – C30:0), ALD (C14, C16), ALK (C9, C23-C31), CH, K, Phy (73.2%), Pri, TMTD.	-	-24.12	-27.11	-2.99	Ruminant
*CW11	357.48	-	SFA (C8:0 – C30:0), MUFA (C16:1, C18:1, C20:1), PUFA (C18:2), BCFA (C14:0 – C27:0), ALD (C18), ALK (C11, C21-C), CH, Phy (73.6%).	-	-26.57	-28.29	-1.72	Ruminant
*CW18	489.48	12.9	SFA (C12:0 – C32:0), MUFA (C16:1, C18:1), PUFA (C18:2), BCFA (C12:0 – C30:0), 10-oxo, D (C8:0 – C13:0), ALD (C18), ALK (C9, C23-C31), HFA (C18), Phy (55.3%), Pri, TMTD.	SFA (C18:0)	-26.23	-28.68	-2.46	Ruminant
*CW20	34.61	-	SFA (C12:0 – C27:0), MUFA (C16:1, C18:1, C22:1), Phy (75.9%).	-	-28.18	-28.62	-0.44	Ruminant
*CW21	34.59	-	SFA (C12:0 – C28:0), MUFA (C16:1, C18:1, C22:1), BCFA (C14:0, C15:0, C17:0, C19:0, C20:0, C21:0, C23:0, C24:0, C25:0, C26:0, C27:0), ALK (C9, C23-C31), CH, ALD (C18), Phy (72%), TMDT.	-	-26.7	-28.45	-1.74	Ruminant
*CW22	436.67	-	SFA (C12:0 – C26:0), MUFA (C16:1, C18:1, C20:1, C22:1), PUFA (C18:2), BCFA (C14:0, C15:0, C17:0, C19:0, C21:0, C22:0), ALK (C16-C31), HFA, Phy (60%), TMDT.	-	-26.86	-30.5	-3.64	Ruminant Dairy
*CW24	87.09	-	SFA (C12:0 – C28:0), MUFA (C16:1, C18:1), PUFA (C18:2), BCFA (C14:0, C15:0, C17:0, C18:0, C19:0), ALK (C11 – C31), 10-oxo, ALD (C18), HFA, Phy (70%), TMDT.	-	-26.3	-28.3	-2	Ruminant
*CW27	23.25	-	SFA (C12:0 – C26:0), MUFA (C16:1, C18:1), BCFA (C14:0 – C18:0), ALK (C23-C31), HFA, Phy (72.3%).	-	-25.9	-28.05	-2.15	Ruminant
**CW10	46.09	85.47	SFA (C14:0 – C26:0), MUFA (C16:1, C18:1, C22:1), BCFA (C15:0, C16:0, C17:0, C18:0,	SFA (C16:0, C17:0, C18:0)	-29.05	-29.28	-0.23	Non-Ruminant

			C19:0, C21:0), ALK (C23-C31), ALD (C18), Phy (70.4%).					
**CW11	732.84	42.83	SFA (C9:0 – C24:0), MUFA (C16:1, C18:1, C22:1), PUFA (C18:2), BCFA (C14:0, C15:0, C17:0, C18:0, C19:0), ALK, HFA, Pri, Phy (68.9%).	SFA (C16:0, C17:0, C18:0)	-29.28	-29.34	-0.05	Non-Ruminant
**CW18	522.13	3.57	SFA (C14:0 – C24:0), MUFA (C16:1, C18:1, C22:1), BCFA (C14:0, C15:0, C17:0, C18:0, C19:0), TMTD, Phy (50.3%).	SFA (C18:0)	-28.46	-29.41	-0.95	Non-Ruminant
**CW20	135.27	9.38	SFA (C12:0, C14:0 – C20:0, C22:0, C23:0), MUFA (C16:1, C18:1, C22:1), BCFA (C15:0, C16:0, C17:0, C18:0).	SFA (C16:0, C18:0)	-29.5	-29.45	0.06	Non-Ruminant
**CW22	2195.88	77.38	SFA (C8:0 – C26:0), MUFA (C16:1, C18:1, C22:1), PUFA (C18:2) BCFA (C14:0, C15:0, C17:0, C18:0) D (C8:0 - C18:0), HFA, TMTD, Phy (58.4%).	SFA (C16:0, C17:0, C18:0)	-27.24	-31.36	-4.13	Ruminant Dairy
**CW24	41.23	-	SFA (C10:0 – C24:0), MUFA (C16:1, C18:1, C22:1), BCFA (C14:0, C15:0, C17:0, C18:0, C19:0), TMTD, Phy (64.4%).	-	-26.9	-28.84	-1.93	Ruminant
***CW10	16.61	-	SFA (C14:0 – C18:0, C20:0, C22:0, C24:0), MUFA (C16:1, C18:1, C22:1), BCFA (C16:0, C17:0, C18:0).	-	-29.15	-29.09	0.06	Non-Ruminant
***CW20	19428.71	-	SFA (C14:0 – C18:0, C20:0, C22:0), MUFA (C16:1, C18:1, C22:1), BCFA (C15:0, C17:0).	-	-29.94	-29.52	0.42	Non-Ruminant
***CW22	142.96	-	SFA (C14:0 – C18:0, C20:0, C22:0), MUFA (C16:1, C18:1, C22:1), BCFA (C15:0, C17:0).	-	-29.36	-29.66	-0.3	Non-Ruminant

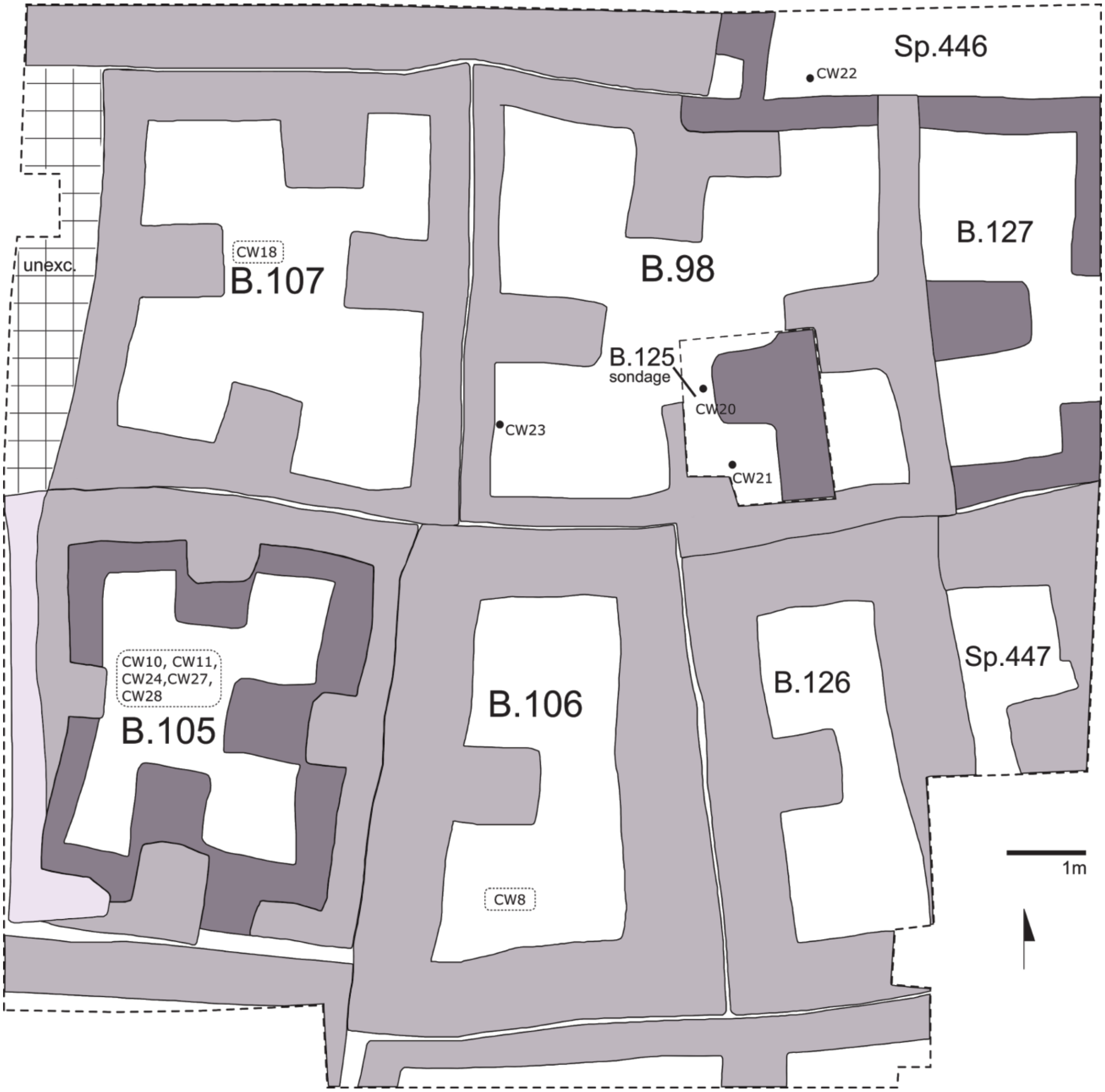

Supplementary Figure 1. Trench locations and context of sherds analyzed in this study. Shades indicate different building phases. Samples associated with dots refer to sherds individually measured with total station, samples enclosed in dotted lines indicate sherds collected from equally well-stratified, yet bulk units of building infill but without individual measurements.

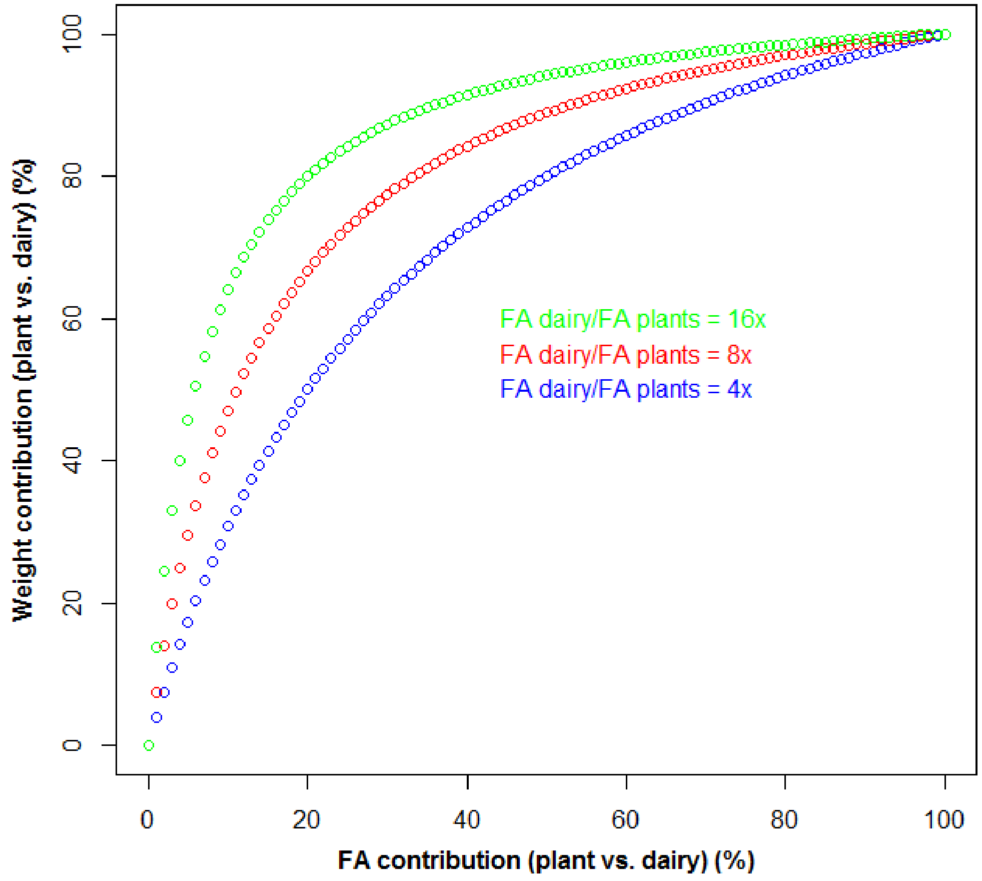

Supplementary Figure 2. Relationship between total weight contributions of plant vs. dairy foods and their FA contributions. Plotted graphs are for different ratios of FA content of dairy vs. plants (4x, 8x, and 16x).

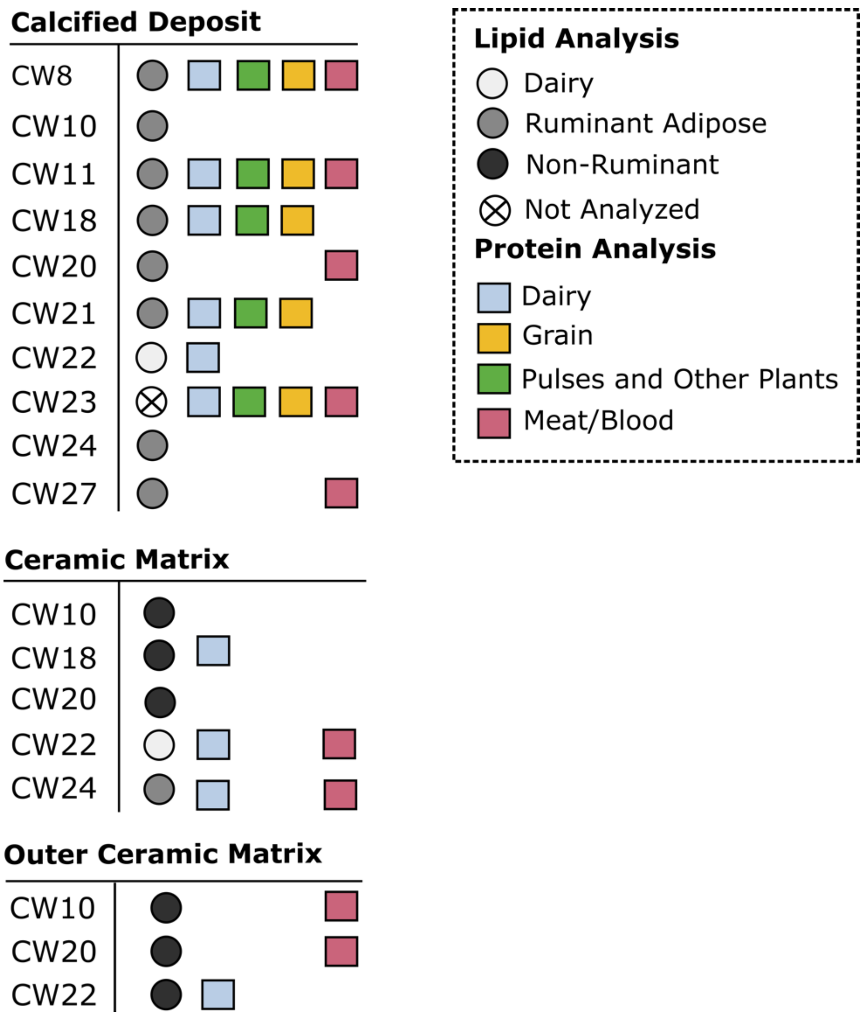

Supplementary Figure 3. Comparison of summarized results from lipid and protein analysis on ceramic matrix and calcified deposit samples.

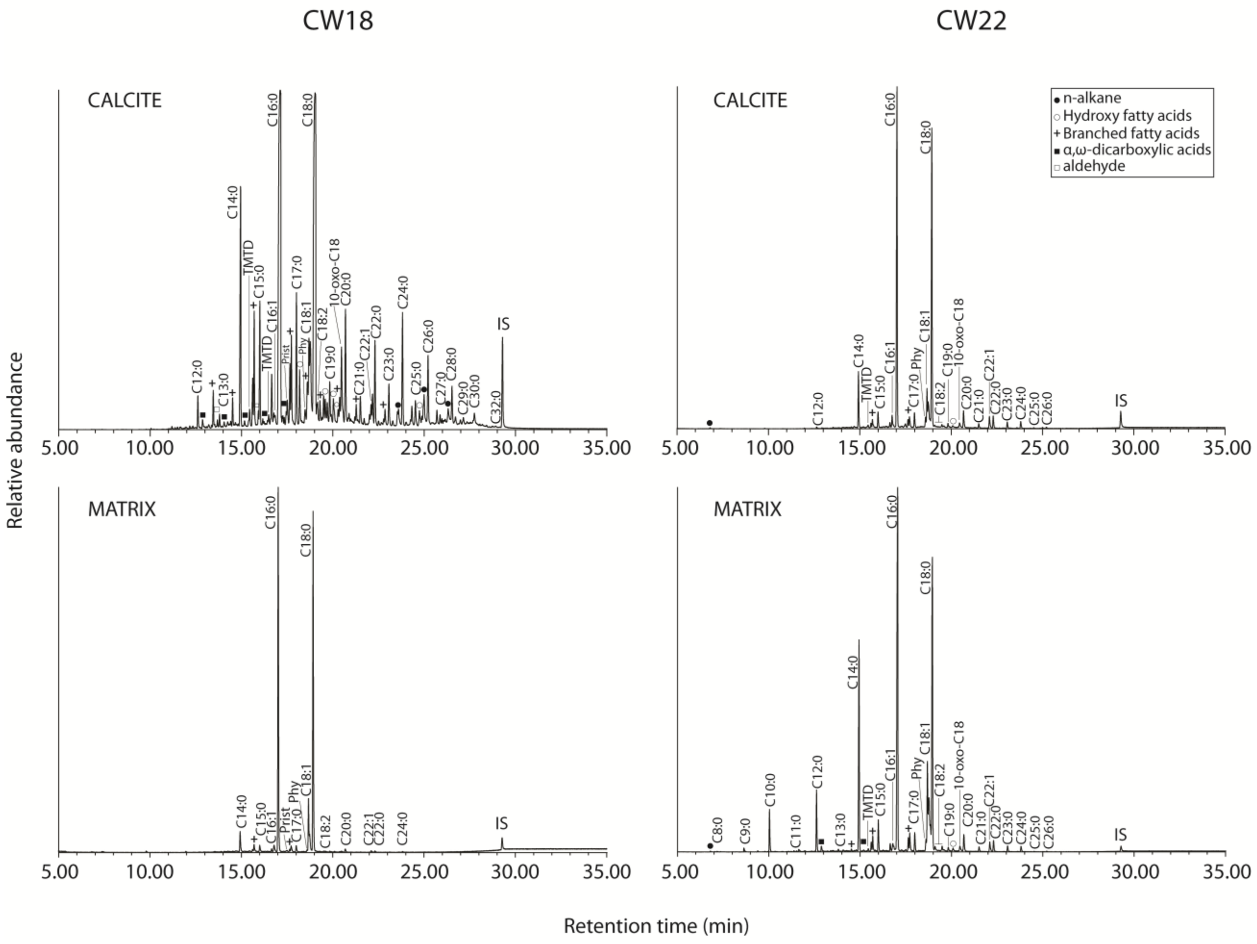

Supplementary Figure 4. Partial total ion chromatogram of typical lipid extracts from calcite (CW18, CW22) and ceramic matrices (CW18 and CW22). Cn:x indicates fatty acid with n carbon atoms and x double bonds. Phy. - phytanic acid; TMTD - 4,8,12-trimethyltridecanoic acid; Pri - pristanic acid; 10-oxo - 10-oxo-octadecanoic acid. IS indicates internal standard.

Supplementary References

1. Lucquin, A., Colonese, A. C., Farrell, T. F. G. & Craig, O. E. Utilising phytanic acid diastereomers for the characterisation of archaeological lipid residues in pottery samples. *Tetrahedron Lett.* **57,** 703–707 (2016).
