# Supplementary material for: Ancient proteins from ceramic vessels at Çatalhöyük West reveal the hidden cuisine of early farmers
Source: Nat Commun. 2018 Oct 3;9:4064. doi: 10.1038/s41467-018-06335-6 (PMC6170438; doi:10.1038/s41467-018-06335-6)
Supplement: Supplementary file 3 — Description of Additional Supplementary Files [file 41467_2018_6335_MOESM3_ESM.pdf]

Description of Additional Supplementary Files

File Name: Supplementary Data 1
Description: Sample metadata, including archaeological context information, key features of ceramic samples and ProteomeXchange metadata

File Name: Supplementary Data 2
Description: Dietary protein and peptide identifications

File Name: Supplementary Data 3
Description: All identified peptides, with “OXF” indicating results from Protein Analysis #1 and “CPN” indicating results from Protein Analysis 2

File Name: Supplementary Data 4
Description: Summary of Zooarchaeology by Mass Spectrometry (ZooMS) data from Çatalhöyük West
